# PLAZA 3.0: an access point for plant comparative genomics

**DOI:** 10.1093/nar/gku986

**Published:** 2014-10-16

**Authors:** Sebastian Proost, Michiel Van Bel, Dries Vaneechoutte, Yves Van de Peer, Dirk Inzé, Bernd Mueller-Roeber, Klaas Vandepoele

**Affiliations:** 1 University of Potsdam, Institute of Biochemistry and Biology, Karl-Liebknecht-Straße 24-25, Haus 20, 14476 Potsdam-Golm, Germany; 2 Max-Planck Institute of Molecular Plant Physiology, Am Mühlenberg 1, 14476 Potsdam-Golm, Germany; 3 VIB, Department of Plant Systems Biology, Technologiepark 927, Ghent, Belgium; 4 Department of Plant Biotechnology and Bioinformatics, Ghent University, Technologiepark 927, Ghent, Belgium; 5 Genomics Research Institute (GRI), University of Pretoria, Private bag X20, Pretoria, 0028, South Africa

## Abstract

Comparative sequence analysis has significantly altered our view on the complexity of genome organization and gene functions in different kingdoms. PLAZA 3.0 is designed to make comparative genomics data for plants available through a user-friendly web interface. Structural and functional annotation, gene families, protein domains, phylogenetic trees and detailed information about genome organization can easily be queried and visualized. Compared with the first version released in 2009, which featured nine organisms, the number of integrated genomes is more than four times higher, and now covers 37 plant species. The new species provide a wider phylogenetic range as well as a more in-depth sampling of specific clades, and genomes of additional crop species are present. The functional annotation has been expanded and now comprises data from Gene Ontology, MapMan, UniProtKB/Swiss-Prot, PlnTFDB and PlantTFDB. Furthermore, we improved the algorithms to transfer functional annotation from well-characterized plant genomes to other species. The additional data and new features make PLAZA 3.0 (http://bioinformatics.psb.ugent.be/plaza/) a versatile and comprehensible resource for users wanting to explore genome information to study different aspects of plant biology, both in model and non-model organisms.

## INTRODUCTION

Since the introduction of next generation sequencing technologies, the price for sequencing a new genome has dropped considerably. While in the past almost exclusively genomes from model organisms were sequenced, the decrease in costs has allowed numerous other plant species with agricultural, economic, environmental or evolutionary importance to be sequenced more recently (1). As sequencing genomic DNA has become accessible to a wide range of researchers, many challenges related to the subsequent data analysis remain, especially for species with large genomes or lacking resources to facilitate genome analysis. The extraction of biological knowledge from a genome sequence, through the detection of similarities and differences with genomes of closely or more distantly related species, is an important concept. By using such comparative approaches, (i) knowledge can be transferred from model to non-model organisms (2), (ii) insights can be gained in the evolution of specific genes or entire metabolic and signaling pathways (3), (iii) genes of importance for niche-specific plant adaptations can be identified (4) and (iv) large-scale genomic events, such as whole-genome duplications (WGDs), can be unveiled (5). As the number of potential pairwise comparisons grows superlinearly with the number of available genomes, such comparative analyses require considerable computational resources. Furthermore, the increase in data poses challenges for efficient storage and retrieval of data, as well as the visualization of data in an accessible and human-interpretable way. Therefore, integrating genomic data from multiple species to generate new biological insights through comparative genomics remains important and challenging.

To overcome these issues, several online comparative genomics platforms are available, each focusing on a specific set of organisms and features. Genome browsers give a detailed representation of the genomic sequence and associated features such as annotated genes, RNA-seq reads, chromatin modifications, etc. (6–8). While such platforms offer a detailed view of a single genome, comparative information is often limited and difficult to interpret in a multispecies context. Platforms focusing on gene families rely on grouping homologous (derived from a common ancestor) genes (9) and within a family detailed phylogenetic reconstructions are possible (10). Less common are tools that look at genes in their genomic context to study cross-species genome evolution and WGDs (11). Finally, comprehensive platforms were created (12–16) which, in contrast to genome browsers, integrate numerous types of information (e.g. gene families, phylogenetic trees and genomic homology) along with structural and functional annotation, providing a versatile starting point for numerous types of analyses, going from simple sequence retrieval over exploring genomic variation to tracing the effects of large-scale duplications.

In this manuscript, we present version 3.0 of PLAZA (http://bioinformatics.psb.ugent.be/plaza/), an online resource that offers comparative genomics data for 37 plant species (Supplementary Table S1) and allows users to browse the annotated genomes, gene families and phylogenetic trees. Furthermore, functional annotation has been transferred from model to non-model organisms using a novel approach, enabling the identification of specific genes or pathways across organisms. Genome organization can be explored through different visualization tools based on gene collinearity or synteny information. The PLAZA Workbench makes it possible for users to analyze multiple genes, stored in an experiment, efficiently, while bulk downloads are available for expert users to perform customized large-scale analyses.

## OVERVIEW AND ACCESS

PLAZA 3.0 has been divided into a monocot- and dicot-centric section containing 31 and 16 species, respectively. This allows the total number of species included in one platform to remain small enough to perform fast searches, load pages quickly and provide responsive visualizations. Both databases contain 10 shared organisms, which either serve as reference species to link between both sections or as outgroups. For each of the included species, the genome sequence and structural annotation has been included along with functional annotation such as Gene Ontology (GO) (17), MapMan (18) and InterPro protein domains (19). While PLAZA can simply act as a browser for such data, the true power of the platform emerges from additional data types generated on top of the original genome information. For instance, homologous genes are grouped together into gene families using BLAST (20) and TribeMCL (21), while subfamilies are identified using BLAST and OrthoMCL (22). For each (sub-)family, multiple sequence alignments are generated and stored that help to unveil conserved protein domains. Pre-computed approximately-maximum-likelihood phylogenetic trees generated using FastTree (23) allow users to explore orthologous and paralogous relations between genes in detail. Based on the phylogenetic trees and (sub-)families, high-quality functional annotations with experimental support from different model organisms (*Arabidopsis thaliana*, *Solanum lycopersicum* and *Oryza sativa*) are transferred to other species lacking functional annotation. Genome evolution can be visualized and studied through remaining collinear regions (regions with conserved gene content and order), which were pre-computed using i-ADHoRe 3.0 (24) and stored in the database.

**Figure 1.**
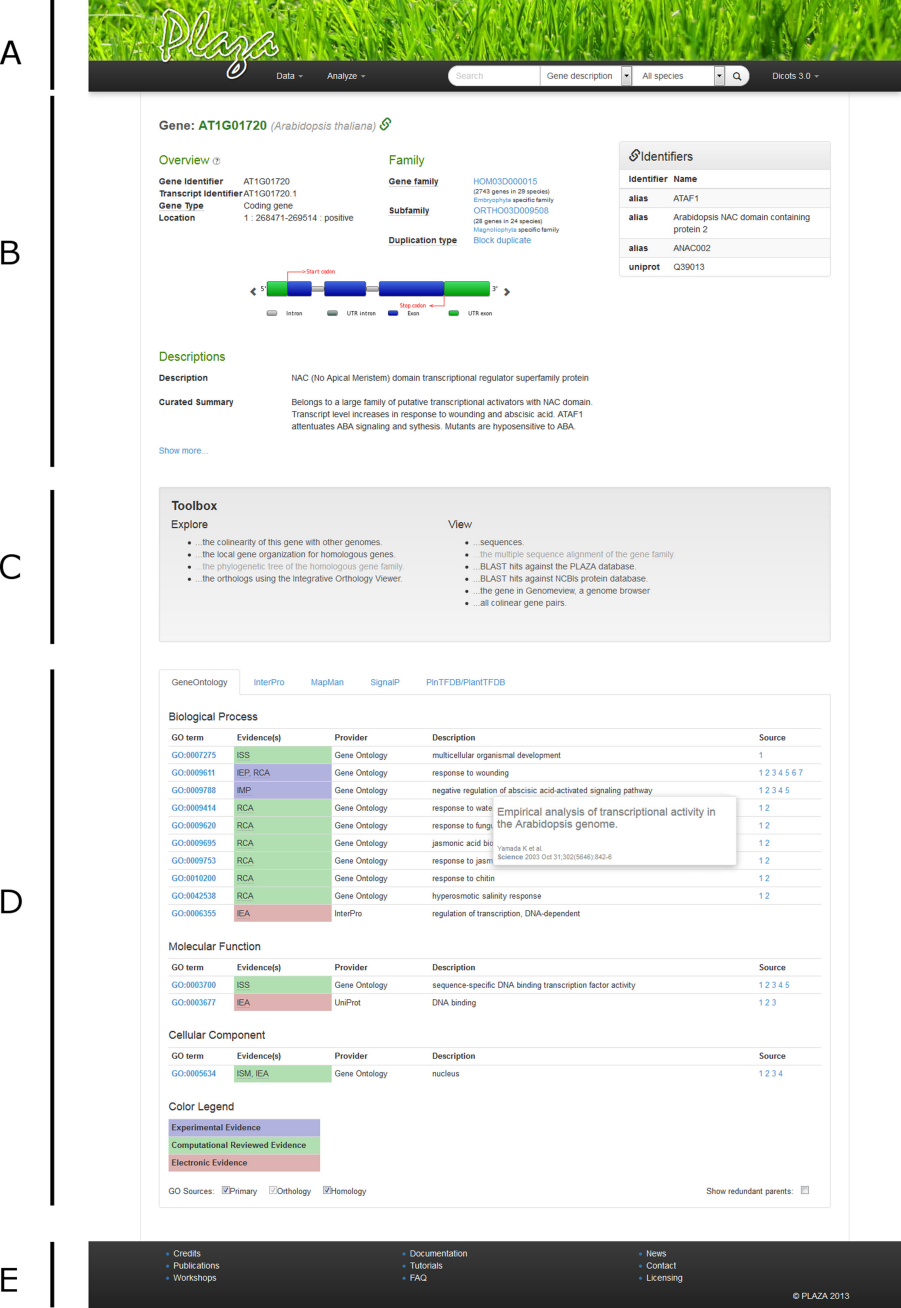
Gene page in PLAZA 3.0. From top to bottom, (**A**) the header with the menu and search functions, (**B**) the general information (links to the gene family, alternative gene names or identifiers) and Descriptions for gene AT1G01720, (**C**) the toolbox that allows different actions to be performed, (**D**) tabs with detailed functional information and (**E**) the footer with links to general information. A similar layout is consistently used on all pages describing different data types.

On the PLAZA portal each data type has its own page, with an intuitive and consistent layout. The top of the page highlights the most general information, with more specific and detailed information further down the page. Numerous hyperlinks are present to allow users to go from one type of data to another (e.g. from a gene to its family or orthologs, or from a gene family to a phylogenetic tree). Every page also has its own toolbox, which provides links to additional analyses and detailed visualizations (Figure 1).

Expert users can download all sequences, gene families, orthology information and functional annotation data in bulk from an FTP server, while the PLAZA Workbench enables the efficient retrieval of sequence or functional information for a set of genes. The latter also allows performing additional analyses, such as GO enrichment, which can be used to unravel overrepresented GO categories in a set of genes for any plant species present in the system.

## NEW FEATURES OF PLAZA 3.0

### New species

**Figure 2.**
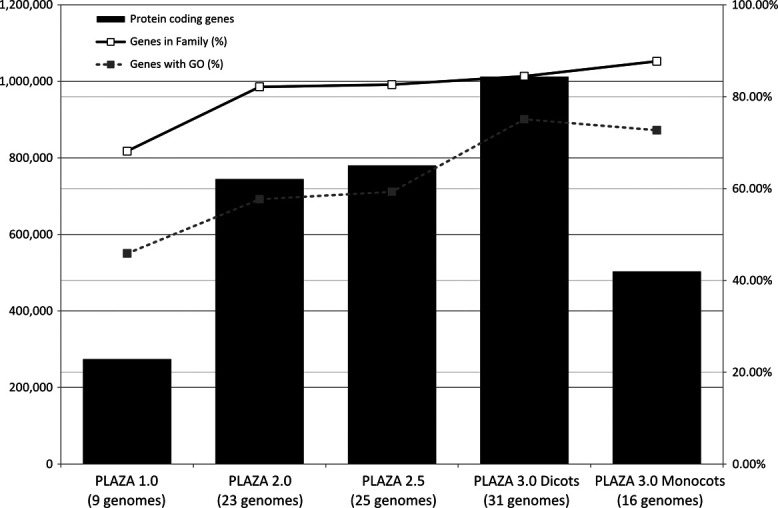
Overview of the number of genes and genomes in the different PLAZA versions. The bar chart (values on the left-axis) indicates the number of genes present in the different PLAZA versions. The superimposed line charts (percentages on the right-axis) denote the percentage of genes present in a gene family or having GO functional annotation. For each version, the number of integrated genomes is shown in parenthesis. Note that there are 10 shared species in PLAZA 3.0 dicots and monocots (Supplementary Table S1).

Currently more than 55 sequenced plant genomes have been released (25), but their quality differs considerably between model organisms that have nearly completed sequences and other species which, so far, were sequenced at low coverage only. The latter are often presented as a collection of small contigs that cannot be assembled into larger scaffolds or ordered into linkage groups or chromosomes. While these low-coverage genomes can be of considerable value in specific studies, the fragmented nature of their sequences results in many partial gene models lacking start or stop codons. A recurring issue with such models is that they hinder the generation of multiple sequence alignments and thus can impair the construction of reliable phylogenetic trees. To avoid such complications, assembly statistics accompanying manuscripts from publically available plant genomes were carefully examined. All genomes that did not meet our quality requirements, based on the N50 number (>500 kb), were excluded. Additionally, in some cases where genomes from closely related species, for instance of the same genus, were available, only the sequence with the highest quality was retained. An overview of the number of genes and species included in the different PLAZA versions is available in Figure 2 and Supplementary Table S1. Note that previous PLAZA releases (14,15,26) will remain available to the scientific community.

#### PLAZA 3.0—Dicots

The majority of novel genomes can be found in the dicots section, with in total 13 new species. Several genomes of plants with economical and agricultural importance are now present in PLAZA, including *Gossypium raimondii* (cotton) (27), *Eucalyptus grandis* (eucalyptus) (28), *Solanum lycopersicum* (tomato) (29), *Solanum tuberosum* (potato) (30), *Beta vulgaris* (sugar beet) (31), *Prunus persica* (peach) (32), *Citrus sinensis* (sweet orange) (33), *Cucumis melo* (melon) (34) and *Citrullus lanatus* (watermelon) (35). In addition to *Arabidopsis thaliana (**36*) and *Arabidopsis lyrata (**37*), which were already included in PLAZA, three new Brassicaceae species (*Capsella rubella* (38), *Brassica rapa* (39) and *Thelungiella parvula* (40)) are now included. Having a large sample of closely related species allows evolutionary biologists to study genomic adaptations to specific niches and how evolution has altered genes and gene families in a recent evolutionary timeframe. An additional distant outgroup species, *Amborella trichopoda* (41), was also included. Amborella is the last remaining member of the Amborellaceae, a sister clade to all other angiosperms, offering unique opportunities to study the diversification of flowering plants and their specific adaptations at the genomic level.

#### PLAZA 3.0 —Monocots

New genomes present in the monocot section of PLAZA 3.0 are *Musa acuminata* (banana) (42), *Setaria italica* (foxtail millet) (43) and *Hordeum vulgare* (barley) (44). All cereals from previous versions remained, though the *Oryza sativa* ssp. japonica (rice) genome was updated to release 7 of MSU Rice Gene Models (45).

### Improved functional annotation

In the previous versions of PLAZA, GO was used to assign Cellular Components, Molecular Functions and Biological Processes to genes, and InterPro domains (19) were included to indicate the functional regions of encoded proteins. Both these types remain in PLAZA 3.0, but in addition MapMan (18) has been included as an additional ontology to describe gene functions. MapMan was initially designed for *Arabidopsis thaliana*, but has recently been applied to other plants as well. Transcription factor families are also easier to identify in PLAZA 3.0 as PlnTFDB (46) and PlantTFDB (47) classifications have now been integrated.

As in earlier versions, experimentally confirmed GO annotation was transferred using a stringent, tree-based, orthology projection method (14). For each gene, all orthologs (genes derived from a common ancestor through speciation, considered to have the same function in different organisms) were identified based on a phylogenetic tree following a strict set of rules: (i) bootstrap values of the nodes considered needed to be 0.7 or higher and (ii) to avoid including co-orthologs from distantly related species, tree-based orthologs were limited to either dicots or monocots.

To facilitate the projection of high-quality functional annotation data over greater phylogenetic distances, two new methods were implemented (see Supplementary Method 1 for details). First, the integrative orthology approach (iOrtho), where four different methods to detect orthologs (using a BLAST-, clustering-, tree- and collinearity-based approach) are combined into a single prediction (15), is now used to transfer functional annotation from species with experimental evidence (Arabidopsis, tomato and rice) to all other species. While this allows transfer over greater evolutionary distances, the use of multiple methods assures that GO terms are only assigned to genes that are confirmed by multiple orthology inference approaches, avoiding potential overprediction. Second, we included a method based on homologous gene families, where enriched functional terms (i.e. GO terms that occur in a family significantly more often than in the whole database and cover at least 50% of the family members having primary GO annotations) are assigned to all other family members lacking this term.

**Figure 3.**
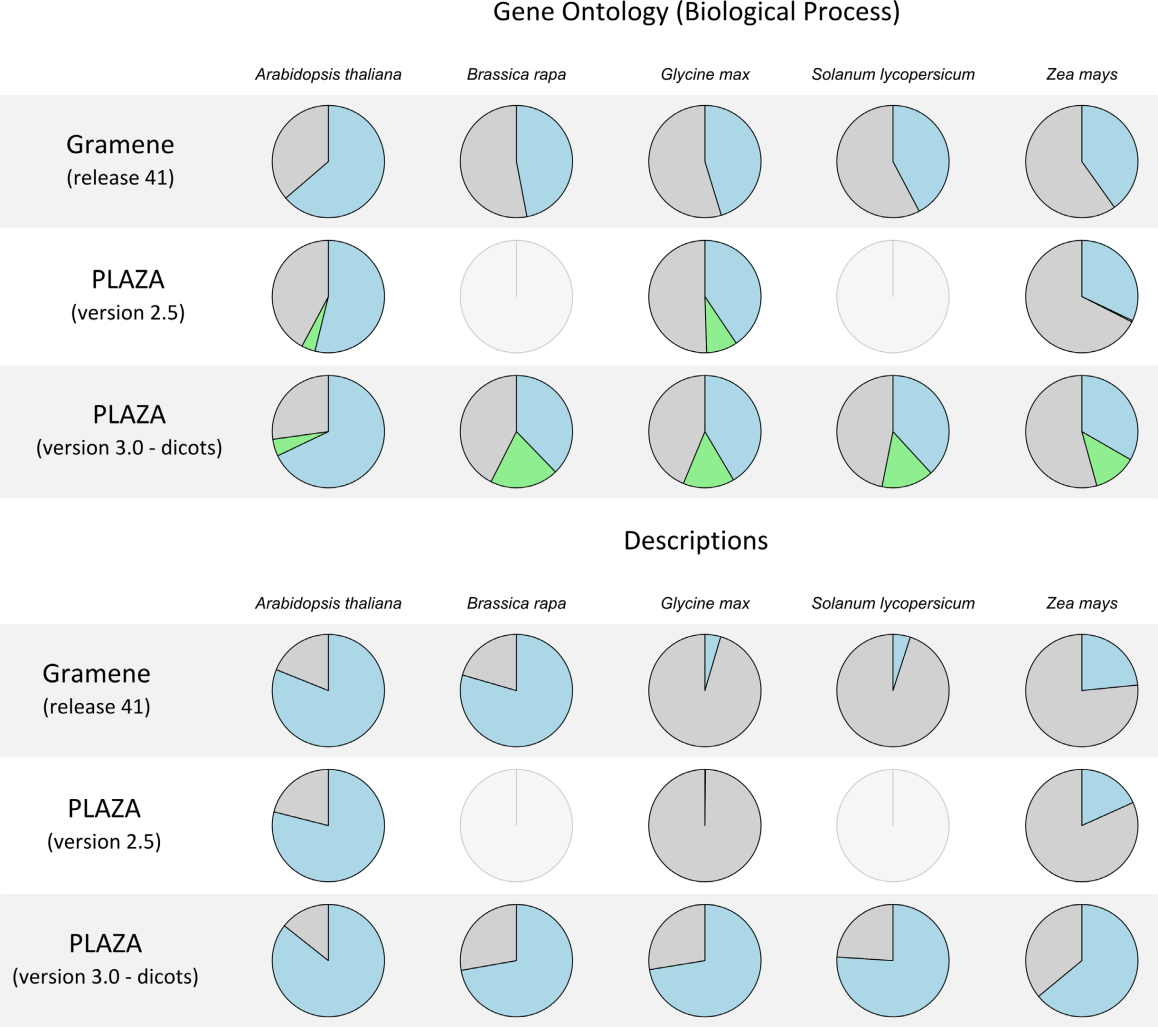
Fractions of genes in Gramene (release 41), PLAZA 2.5 and PLAZA 3.0 dicots with a description and a GO Biological Process annotation for five selected species (*Arabidopsis thaliana*, *Brassica rapa*, *Glycine max*, *Solanum lycopersicum* and *Zea mays*). Blue indicates the fraction with a description or primary GO label (derived from the GO consortium, UniProtKB/Swiss-Prot or InterProScan), green the fraction with a projected GO label only and gray the fraction without description/GO.

Figure 3 illustrates the fraction of genes that have a GO Biological Process label provided by the GO consortium, UniProtKB/Swiss-Prot or found using InterProScan (primary source, blue), found using PLAZA 3.0's GO projection (green) or that lack an annotation (gray). While the amount of primary annotation is similar to Gramene (release 41) (16) and PLAZA 2.5, the new GO projection is able to assign a Biological Process to considerably more genes. Especially for *Zea mays* (corn), there is a large improvement as the current method allows information to be transferred over large phylogenetic distances (i.e. from dicots to monocots).

On a gene page, the different sources of functional annotation are displayed and in cases where the annotation was transferred from another gene, the origin and projection method (homology-based, iORTHO or tree-based orthology) used are shown (Supplementary Figure S1). Users have the option to only consider primary labels (from the GO consortium, UniProtKB/Swiss-Prot and InterProScan), to additionally include orthology-based projected terms, or to take all GO annotations into account (primary, orthology-based and homology-based projection).

For the best annotated species (e.g. *Arabidopsis thaliana*), well-curated genes come with short descriptions provided by expert annotators. For species with less extensive annotation, such easily interpretable descriptions are rare or lacking completely. Therefore, AnnoMine was used to generate text descriptions (Supplementary Method 2). This tool performed, for all genes, sequence similarity searches against the UniProtKB/Swiss-Prot (48) database, which contain curated high-quality gene descriptions. Gene descriptions from BLAST hits, weighted by the BLASTP E-value, were processed by an integrative text-mining algorithm that, based on statistically overrepresented co-occurrences of words, assigned a description to the gene.

The fraction of genes that have a description in five selected species is shown in Figure 3. For *Arabidopsis thaliana* extensive annotation efforts assigned descriptions to the majority of the genes and while these efforts have been transferred to closely related Brassicaceae species, for more distant species proper descriptions are often lacking. In contrast with other platforms and earlier PLAZA releases, now a large fraction of genes have an AnnoMine gene description (63% of the dicot and monocot protein-coding genes), including many genes from non-model plants. Although this text-mining procedure cannot replace expert annotators, it provides a valuable functional indication in the absence of a curated description.

### Genome evolution

Collinearity, defined as conservation of gene content and order, has been used in PLAZA to determine homologous regions between genomes and duplicated regions within a genome. The latter are usually remnants of large-scale duplication events and various studies have revealed that traces of WGDs are present in all plant genomes sequenced to date (49). However, as gene loss and rearrangements accumulate after such an event, the detection of WGDs using collinearity becomes increasingly difficult as their ages increase (50). Therefore, in some cases, collinearity is a suboptimal measure to detect remnants of ancient duplications. To overcome this limitation, PLAZA 3.0 now also includes information on syntenic duplicates, which are paralogs from regions with conserved gene content regardless of the order (51). As such, an additional 125 266 and 55 277 genes were found to be putatively derived from WGDs that were not found by the default collinearity searches in the dicots and monocots versions, respectively (Supplementary Method 3).

### Technical improvements

While not directly visible for users, considerable changes have been made to build the PLAZA 3.0 platform and store the different data types. Structural changes to the database and the way data are stored now allow faster retrieval, also for complex queries comprising multiple data types. The result is that, despite the increase in data, many pages on the website load faster. For visualizations that summarize large amounts of data (like the Skyline plot, to browse for a locus or region collinearity in multiple species), these improvements resulted in a 2- to 3-fold speed-up.

Furthermore, third-party tools required to build PLAZA have been updated to their latest version or replaced by more modern alternatives. BLAST (20), used to find similarities between proteins prior to gene family delineation, has been upgraded from version 2.2.17 to 2.2.27+, OrthoMCL 1.4 (22) was changed to version 2.0 and InterProScan (52) version 4.6 was replaced with 5.44. In previous builds, two multiple sequence alignment algorithms were used, namely MUSCLE for the alignments shown on the website and ClustalW for calculations of *K*_S_ (the fraction of synonymous substitutions per synonymous site) values. Now MUSCLE, which offers an excellent compromise between speed and accuracy, is used consistently. To further reduce the amount of computing power needed to build PLAZA 3.0, FastTree 2.1.7 (23) was selected to replace PhyML (53) for the construction of phylogenetic trees.

More noticeable for users is that all graphs, which previously were rendered using Flash, were replaced by Javascripts generating SVG output. This has several advantages, such as (i) devices where Flash is not available now will be able to display these graphs and (ii) SVGs can easily be downloaded and stored for future reference or used as high-resolution images for publications. This in combination with a new fluid grid layout (where elements can move position if the necessary monitor width is not available, avoiding the need for horizontal scroll bars) provides excellent support for mobile devices, which are being used by a growing number of visitors. Finally, GenomeView (8), which used to be a java applet started within the browser, has been updated and is now a web-started java application that is considerably faster than previous versions.

## CONCLUSION

PLAZA 3.0 offers an important update toward new publicly available plant genomes while technical improvements result in a web-based portal that loads faster and remains responsive despite the increase in data. A new layout provides a richer, more intuitive user experience while supporting additional devices. Furthermore, through the integration of additional functional classification systems as well as the implementation of new transfer methods, PLAZA 3.0 now offers comprehensive functional annotation for all species included.

## SUPPLEMENTARY DATA

Supplementary Data are available at NAR Online.

## References

[1] Van de Peer Y., Pires J.C. (2012). Getting up to speed. Curr. Opin. Plant Biol..

[2] Leeggangers H.A., Moreno-Pachon N., Gude H., Immink R.G. (2013). Transfer of knowledge about flowering and vegetative propagation from model species to bulbous plants. Int. J. Dev. Biol..

[3] Hamel L.P., Sheen J., Seguin A. (2014). Ancient signals: comparative genomics of green plant CDPKs. Trends Plant Sci..

[4] Tian C.F., Zhou Y.J., Zhang Y.M., Li Q.Q., Zhang Y.Z., Li D.F., Wang S., Wang J., Gilbert L.B., Li Y.R. (2012). Comparative genomics of rhizobia nodulating soybean suggests extensive recruitment of lineage-specific genes in adaptations. Proc. Natl Acad. Sci. U.S.A..

[5] Kim C., Wang X., Lee T.H., Jakob K., Lee G.J., Paterson A.H. (2014). Comparative analysis of Miscanthus and Saccharum reveals a shared whole-genome duplication but different evolutionary fates. Plant Cell.

[6] Stein L.D. (2013). Using GBrowse 2.0 to visualize and share next-generation sequence data. Brief. Bioinform..

[7] Karolchik D., Barber G.P., Casper J., Clawson H., Cline M.S., Diekhans M., Dreszer T.R., Fujita P.A., Guruvadoo L., Haeussler M. (2014). The UCSC Genome Browser database: 2014 update. Nucleic Acids Res..

[8] Abeel T., Van Parys T., Saeys Y., Galagan J., Van de Peer Y. (2012). GenomeView: a next-generation genome browser. Nucleic Acids Res..

[9] Rouard M., Guiga V., Aluome C., Laporte M.A., Droc G., Walde C., Zmasek C.M., Perin C., Conte M.G. (2011). GreenPhylDB v2.0: comparative and functional genomics in plants. Nucleic Acids Res..

[10] Powell S., Forslund K., Szklarczyk D., Trachana K., Roth A., Huerta-Cepas J., Gabaldon T., Rattei T., Creevey C., Kuhn M. (2014). eggNOG v4.0: nested orthology inference across 3686 organisms. Nucleic Acids Res..

[11] Lee T.H., Tang H., Wang X., Paterson A.H. (2013). PGDD: a database of gene and genome duplication in plants. Nucleic Acids Res..

[12] Flicek P., Amode M.R., Barrell D., Beal K., Billis K., Brent S., Carvalho-Silva D., Clapham P., Coates G., Fitzgerald S. (2014). Ensembl 2014. Nucleic acids research.

[13] Lyons E., Pedersen B., Kane J., Alam M., Ming R., Tang H., Wang X., Bowers J., Paterson A., Lisch D. (2008). Finding and comparing syntenic regions among Arabidopsis and the outgroups papaya, poplar, and grape: CoGe with rosids. Plant Physiol..

[14] Proost S., Van Bel M., Sterck L., Billiau K., Van Parys T., Van de Peer Y., Vandepoele K. (2009). PLAZA: a comparative genomics resource to study gene and genome evolution in plants. Plant Cell.

[15] Van Bel M., Proost S., Wischnitzki E., Movahedi S., Scheerlinck C., Van de Peer Y., Vandepoele K. (2012). Dissecting plant genomes with the PLAZA comparative genomics platform. Plant Physiol..

[16] Monaco M.K., Stein J., Naithani S., Wei S., Dharmawardhana P., Kumari S., Amarasinghe V., Youens-Clark K., Thomason J., Preece J. (2014). Gramene 2013: comparative plant genomics resources. Nucleic Acids Res..

[17] Reference Genome Group of the Gene Ontology Consortium (2009). The Gene Ontology's Reference Genome Project: a unified framework for functional annotation across species. PLoS Comput. Biol..

[18] Thimm O., Blasing O., Gibon Y., Nagel A., Meyer S., Kruger P., Selbig J., Muller L.A., Rhee S.Y., Stitt M. (2004). MAPMAN: a user-driven tool to display genomics data sets onto diagrams of metabolic pathways and other biological processes. Plant J..

[19] Hunter S., Jones P., Mitchell A., Apweiler R., Attwood T.K., Bateman A., Bernard T., Binns D., Bork P., Burge S. (2012). InterPro in 2011: new developments in the family and domain prediction database. Nucleic Acids Res..

[20] Altschul S.F., Madden T.L., Schaffer A.A., Zhang J., Zhang Z., Miller W., Lipman D.J. (1997). Gapped BLAST and PSI-BLAST: a new generation of protein database search programs. Nucleic Acids Res..

[21] Enright A.J., Van Dongen S., Ouzounis C.A. (2002). An efficient algorithm for large-scale detection of protein families. Nucleic Acids Res..

[22] Li L., Stoeckert C.J., Roos D.S. (2003). OrthoMCL: identification of ortholog groups for eukaryotic genomes. Genome Res..

[23] Karimi M., Inze D., Depicker A. (2002). GATEWAY vectors for Agrobacterium-mediated plant transformation. Trends Plant Sci..

[24] Proost S., Fostier J., De Witte D., Dhoedt B., Demeester P., Van de Peer Y., Vandepoele K. (2012). i-ADHoRe 3.0—fast and sensitive detection of genomic homology in extremely large data sets. Nucleic Acids Res..

[25] Michael T.P., Jackson S. (2013). The first 50 plant genomes. Plant Genome.

[26] Vandepoele K., Van Bel M., Richard G., Van Landeghem S., Verhelst B., Moreau H., Van de Peer Y., Grimsley N., Piganeau G. (2013). pico-PLAZA, a genome database of microbial photosynthetic eukaryotes. Environ. Microbiol..

[27] Paterson A.H., Wendel J.F., Gundlach H., Guo H., Jenkins J., Jin D., Llewellyn D., Showmaker K.C., Shu S., Udall J. (2012). Repeated polyploidization of Gossypium genomes and the evolution of spinnable cotton fibres. Nature.

[28] Myburg A.A., Grattapaglia D., Tuskan G.A., Hellsten U., Hayes R.D., Grimwood J., Jenkins J., Lindquist E., Tice H., Bauer D. (2014). The genome of Eucalyptus grandis. Nature.

[29] The Tomato Genome Consortium (2012). The tomato genome sequence provides insights into fleshy fruit evolution. Nature.

[30] Xu X., Pan S., Cheng S., Zhang B., Mu D., Ni P., Zhang G., Yang S., Li R., Wang J. (2011). Genome sequence and analysis of the tuber crop potato. Nature.

[31] Dohm J.C., Minoche A.E., Holtgrawe D., Capella-Gutierrez S., Zakrzewski F., Tafer H., Rupp O., Sorensen T.R., Stracke R., Reinhardt R. (2014). The genome of the recently domesticated crop plant sugar beet (Beta vulgaris). Nature.

[32] Peach Genome Initiative International, Verde I., Abbott A.G., Scalabrin S., Jung S., Shu S., Marroni F., Zhebentyayeva T., Dettori M.T., Grimwood J. (2013). The high-quality draft genome of peach (Prunus persica) identifies unique patterns of genetic diversity, domestication and genome evolution. Nat. Genet..

[33] Xu Q., Chen L.L., Ruan X., Chen D., Zhu A., Chen C., Bertrand D., Jiao W.B., Hao B.H., Lyon M.P. (2012). The draft genome of sweet orange (Citrus sinensis). Nat. Genet..

[34] Garcia-Mas J., Benjak A., Sanseverino W., Bourgeois M., Mir G., Gonzalez V.M., Henaff E., Camara F., Cozzuto L., Lowy E. (2012). The genome of melon (Cucumis melo L.). Proc. Natl Acad. Sci. U.S.A..

[35] Guo S., Zhang J., Sun H., Salse J., Lucas W.J., Zhang H., Zheng Y., Mao L., Ren Y., Wang Z. (2013). The draft genome of watermelon (Citrullus lanatus) and resequencing of 20 diverse accessions. Nat. Genet..

[36] Arabidopsis Genome Initiative (2000). Analysis of the genome sequence of the flowering plant Arabidopsis thaliana. Nature.

[37] Hu T.T., Pattyn P., Bakker E.G., Cao J., Cheng J.F., Clark R.M., Fahlgren N., Fawcett J.A., Grimwood J., Gundlach H. (2011). The Arabidopsis lyrata genome sequence and the basis of rapid genome size change. Nat. Genet..

[38] Slotte T., Hazzouri K.M., Agren J.A., Koenig D., Maumus F., Guo Y.L., Steige K., Platts A.E., Escobar J.S., Newman L.K. (2013). The Capsella rubella genome and the genomic consequences of rapid mating system evolution. Nature genetics.

[39] Wang X., Wang H., Wang J., Sun R., Wu J., Liu S., Bai Y., Mun J.H., Bancroft I., Cheng F. (2011). The genome of the mesopolyploid crop species Brassica rapa. Nat. Genet..

[40] Dassanayake M., Oh D.H., Haas J.S., Hernandez A., Hong H., Ali S., Yun D.J., Bressan R.A., Zhu J.K., Bohnert H.J. (2011). The genome of the extremophile crucifer Thellungiella parvula. Nat. Genet..

[41] Amborella Genome Project (2013). The Amborella genome and the evolution of flowering plants. Science.

[42] D'Hont A., Denoeud F., Aury J.M., Baurens F.C., Carreel F., Garsmeur O., Noel B., Bocs S., Droc G., Rouard M. (2012). The banana (Musa acuminata) genome and the evolution of monocotyledonous plants. Nature.

[43] Bennetzen J.L., Schmutz J., Wang H., Percifield R., Hawkins J., Pontaroli A.C., Estep M., Feng L., Vaughn J.N., Grimwood J. (2012). Reference genome sequence of the model plant Setaria. Nat. Biotechnol..

[44] Mayer K.F., Waugh R., Brown J.W., Schulman A., Langridge P., Platzer M., Fincher G.B., Muehlbauer G.J., Sato K., International Barley Genome Sequencing Consortium (2012). A physical, genetic and functional sequence assembly of the barley genome. Nature.

[45] International Rice Genome Sequencing Project (2005). The map-based sequence of the rice genome. Nature.

[46] Perez-Rodriguez P., Riano-Pachon D.M., Correa L.G., Rensing S.A., Kersten B., Mueller-Roeber B. (2010). PlnTFDB: updated content and new features of the plant transcription factor database. Nucleic Acids Res..

[47] Jin J., Zhang H., Kong L., Gao G., Luo J. (2013). PlantTFDB 3.0: a portal for the functional and evolutionary study of plant transcription factors. Nucleic Acids Res..

[48] UniProt Consortium (2014). Activities at the Universal Protein Resource (UniProt). Nucleic Acids Res..

[49] Vanneste K., Maere S., Van de Peer Y. (2014). Tangled up in two: a burst of genome duplications at the end of the Cretaceous and the consequences for plant evolution. Philos. Trans. R. Soc. Lond. B Biol. Sci..

[50] Van de Peer Y., Fawcett J.A., Proost S., Sterck L., Vandepoele K. (2009). The flowering world: a tale of duplications. Trends Plant Sci..

[51] Ruelens P., de Maagd R.A., Proost S., Theissen G., Geuten K., Kaufmann K. (2013). FLOWERING LOCUS C in monocots and the tandem origin of angiosperm-specific MADS-box genes. Nat. Commun..

[52] Quevillon E., Silventoinen V., Pillai S., Harte N., Mulder N., Apweiler R., Lopez R. (2005). InterProScan: protein domains identifier. Nucleic Acids Res..

[53] Guindon S., Gascuel O. (2003). A simple, fast, and accurate algorithm to estimate large phylogenies by maximum likelihood. Syst. Biol..

